# Optimization of adefovir therapy in chronic hepatitis B according to baseline predictors and on-treatment HBV DNA: a 5-Year prospective study

**DOI:** 10.1186/1743-422X-8-444

**Published:** 2011-09-21

**Authors:** Hui Lu, Da Ying Geng, Fei Shen, Jing Yao Zhang, Bing Lu, Li Xian Ma

**Affiliations:** 1 Jinan Infectious Disease Hospital, affiliated to Shandong University, Jinan, China; 2 Department of Infectious Diseases, Qilu Hospital of Shandong University, Jinan, China; 3 Brigham & Women's Hospital, Harvard Medical School, Boston, MA, USA

**Keywords:** hepatitis B, chronic, Adefovir Dipivoxil, therapy, hepatitis B virus, hepatitis Be antigen positive, predictor

## Abstract

**Background:**

Adefovir Dipivoxil (ADV) is an important agent to suppress hepatitis B virus (HBV) replication with suboptimal effect on virological and serological response. To optimize Adefovir therapy in chronic hepatitis B (CHB) patients with hepatitis B e antigen (HBeAg) positive, we studied the baseline parameters and on-treatment HBV DNA for favorable outcomes.

**Methods:**

48 patients were enrolled in the study and followed up for 5 years prospectively. Baseline characteristics, virological, serological and biochemical parameters as well as on treatment HBV DNA were assessed in prediction of favorable outcomes.

**Results:**

1. The patients with baseline alanine aminotransferase (ALT) ≥5 × the upper limit of normal (ULN, 40 IU/L) had higher rates of viral response (VR), HBeAg loss and HBeAg seroconversion at year 5 compared to the patients with ALT < 5 × ULN (VR: 75% vs 43.8%, p = 0.035; HBeAg loss: 43.9% vs 13.8%, p = 0.017; HBeAg seroconversion: 37.9% vs 13.8%, p = 0.035); Patients with baseline HBV DNA < 10^9 ^copies/ml and ALT ≥3 × ULN had more chance of HBeAg seroconversion (40.9% vs 8.7%, p = 0.012), while in patients with HBeAg < 800 s/co or HBsAg < 5000 IU/ml higher rates of HBeAg loss were achieved. 2. HBV DNA level < 10^4 ^copies/ml at week 24 was predictive for VR (96.0% vs 40.9%, P < 0.001), HBeAg loss (84.0% vs 36.3%, P = 0.001) and HBeAg seroconversion (36.0% vs 9.1%, P = 0.030).

**Conclusions:**

ADV treatment should be started for patients with baseline ALT≥5 × ULN or patients with ALT≥3 × ULN and HBV DNA < 10^9 ^copies/ml. Lower level of HBeAg(< 800 s/co) and HBsAg(< 5000 IU/ml) may be regarded as referenced factors. In patients with serum HBV DNA < 10^4 ^copies/ml at week 24 the therapy should continue, and a favorable outcome may be achieved in 5 years or longer.

## Background

Adefovir Dipivoxil (ADV) is a nucleotide analogue for treatment of hepatitis B. Long term treatment with ADV is well tolerated and may produce long-term virological, biochemical, serological and histological improvement [1]. Compared with lamivudine (LAM) and telbivudine, ADV correlated resistance develops less frequently but ADV has less potent activity against hepatitis B virus (HBV). It has been reported that the rates of HBV DNA negative, hepatitis B e antigen (HBeAg) loss and HBeAg seroconversion were 39%, 58% and 48% respectively in the subjects with HBeAg positive chronic hepatitis B (CHB) treated with ADV 10 mg once daily for 5 years, and 66% of them achieved alanine aminotransferase (ALT) normalization and 20% developped HBV mutant [2]. These data highlighted an urgent need to identify the predictors that will help to find candidates who may likely benefit from ADV or, alternatively, determine whether other extensive treatment is needed. Recently, It has been reported that baseline and on-treatment serum HBV DNA were significant predictors for sustained viral response in CHB patients with pegylated interferon(Peg-IFN) and nucleos(t)ide analogues(NAs) treatment [3-8]. In the GLOBE study of HBeAg-positive patients treated with telbivudine, HBV DNA negative at week 24 was more predictive for responses than other parameters [9]. Similar results were reported in LAM and ADV therapies [10,11]. However, the association of these predictors with effect of long term ADV treatment has rarely been reported.

The aim of this study is to assess baseline and on-treatment serum markers in prediction of long term response in the early phase of treatment with ADV among HBeAg-positive CHB patients so as to identify the most beneficial patients from ADV treatment and optimize the therapy.

## Methods

### Subject population

48 HBeAg-positive CHB patients who enrolled in the study were treated with ADV (provided by GlaxoSmithKline) and followed up for 5 years. All patients were seropositive for hepatitis B surface antigen (HBsAg) and HBeAg for more than 6 months and with baseline ALT ≥1.2 × the upper limit of normal (ULN, 40 IU/L) and HBV DNA ≥10^6 ^copies/ml before treatment. Criteria for exclusion included serum creatinine greater than 1.5 mg/dl (130μmol/L); seropositivity for hepatitis C or D virus or human immunodeficiency virus. No patients received LAM or any other anti-HBV therapy within 6 months prior to treatment. Other treatments were not used during the therapy. Written informed consent was obtained from all patients and the study was approved by the Ethical Committee of Jinan Infectious Disease Hospital.

### Study Design

All patients were followed up at weeks 4, 12, 24, 52 and every 12-16 weeks after week 52 for detection of serum biochemical parameters, serum viral load (Roche COBAS Amplicore HBV Monitor PCR assay, lower limit of detection 300 copies/ml) and HBV markers (micro particle enzyme immunoassay with ABBOTT reagents). DNA sequencing and phylogenetic analysis of HBV genome were evaluated at baseline. Serum samples at first time point from subjects who experienced a breakthrough of HBV DNA (HBV DNA level increased by 1 log_10 _copies/ml or more than the treatment nadir) were assessed for the development of ADV-associated mutations (rtA181V and rtN236T) in the HBV polymerase.

### Efficacy End Points

The primary efficacy endpoints in this study were HBeAg seroconversion defined as combination of HBeAg loss and development of hepatitis B e antibody accompanied by HBV DNA negative(< 300 copies/ml). The secondary efficacy endpoints are HBeAg loss and viral response (VR) defined as HBV DNA negative.

### Statistical Analyses

Descriptive statistics such as the mean, median, and standard deviation for each continuous variable and frequencies for each categorical variable were used to summarize the data as well as detect outliers and missing values. The associations of baseline and on-treatment parameters with virological, serological and biochemical responses were assessed using Chi-square tests or Fisher's exact tests. All p values were calculated with two-sided significance level of 0.05. Data analyses were performed using SAS 9.2 (SAS Institute, Inc, Cary, North Carolina).

## Results

### 1. Baseline characteristics of demographic, clinical and laboratory data

Except for 1 woman who withdrew because of pregnance at week 104, 47 patients completed 5 years' follow up. All patients were ethnically Chinese and HBV genotype C positive. The baseline demographic, clinical and virological data are shown in Table 1.

**Table 1:** Baseline characteristics of demographic, clinical and laboratory data

variables	
Age in years, mean ± SD	31.7 ± 6.6
Male, %	85.1%
HBV genotype C, %	100%
Baseline ALT, IU/L	216.3 ± 169.7
Baseline HBV DNA(log_10_copies/ml)	8.5 ± 0.8
Baseline HBeAg(log_10_)	2.8 ± 0.4
BaselineHBsAg(log_10_)	3.7 ± 0.5

### 2. Virological, serological and biochemical response

**Figure 1:**
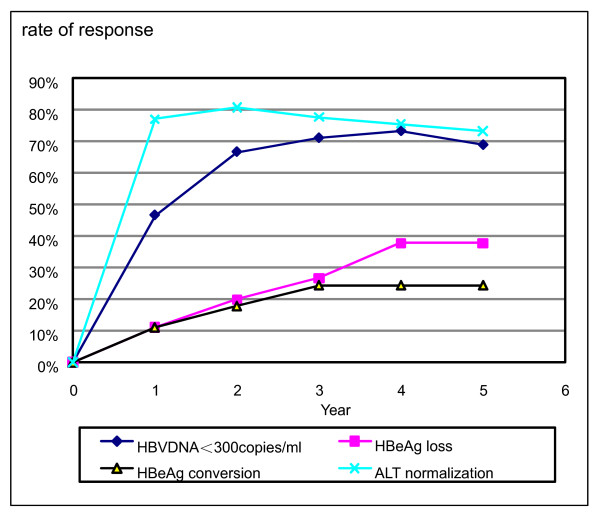
**Virological, serological and biochemical response over time**. The rates of VR, accumulative HBeAg loss, HBeAg seroconversion increased with time.

Of 47 patients, the rates of VR, accumulative HBeAg loss, HBeAg seroconversion and ALT normalization in the end of the fifth year were 66.0%, 36.2%, 23.4% and 70.2%, respectively. The rates of HBeAg loss increased over time, and the proportion of patients with ALT normalization and HBV DNA < 300 copies/ml achieved maximal values after 2 and 4 years respectively (Figure 1). Virus breakthrough was found in 4 patients but ADV correlated variants were detectable only in 3 of them.

### 3. Relationship between baseline parameters and 5-year response

We evaluated baseline characteristics such as ALT, HBV DNA, HBsAg and HBeAg level in prediction of 5-year response and found baseline ALT was a potential predictor of outcome. Patients with baseline ALT ≥5 × ULN were more likely to have HBeAg seroconversion, HBeAg loss and VR than patients with ALT < 5 × ULN (Table 2), while in patients with HBeAg < 800 s/co or HBsAg < 5000 IU/ml higher rates of HBeAg loss were achieved. The baseline HBeAg level predicted VR too, as 88.9% patients with baseline HBeAg < 800 s/co became HBV DNA negative, whereas baseline HBV DNA level alone was not a significant predictor for HBeAg seroconversion, HBeAg loss or VR.

**Table 2:** Baseline predictors and 5-year outcomes

	VR %	P-value	HBeAg loss %	P-value	HBeAg seroconversion %	P-value
ALT≥5 × ULN	75.0%	0.035	43.9%	0.017	37.9%	0.035
ALT < 5 × ULN	43.8%		13.8%		13.8%	
HBeAg < 800 s/co	88.9%	0.027	77.8%	0.045	27.8%	0.831
HBeAg≥800 s/co	47.6%		38.0%		19.0%	
HBsAg < 5000 IU/ml	80.0%	0.132	80.0%	0.026	27.8%	0.200
HBsAg≥ 5000 IU/ml	59.3%		48.1%		24.1%	
HBV DNA < 9 log_10 _copies/ml	71.9%	0.494	61.8%	0.621	26.5%	0.702
HBV DNA≥ 9 log_10_copies/ml	69.2%		53.9%		15.4%	

**Figure 2:**
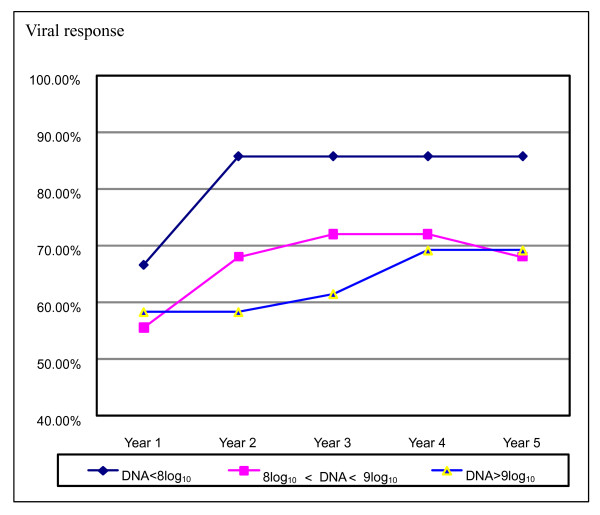
**Viral response in patients with different levels of baseline viral load**. The rate of VR in the 5 years decreased with the higher levels of baseline viral load although no statistical significance was observed.

Even so, there was a trend that the rate of VR in the 5 years decreased with the higher levels of baseline viral load (Figure 2). In subgroup analyses, we found in patients with baseline HBV DNA < 10^9 ^copies/ml, ALT was positively associated with VR and HBeAg seroconversion. The rate of HBeAg seroconversion was significantly higher in patients with ALT ≥3 × ULN when compared to the patients with ALT < 3 × ULN (40.9% vs 8.7%, p = 0.012).

### 4. Impact of on-treatment HBV DNA on outcomes

The relationships between on-treatment HBV DNA levels and 5-year outcomes were presented in Table 3. Patients with serum HBV DNA level < 4 log_10 _copies/ml at week 24 had higher rates of HBeAg seroconversion, HBeAg loss and VR compared to the patients with serum HBV DNA ≥4 log_10 _copies/ml. The drop of the serum HBV DNA ≥3 log_10 _copies/ml at week 4 and HBV DNA undetectable at week 52 can predict VR and HBeAg loss while the drop of the serum HBV DNA ≥4 log_10 _copies/ml at week 12 was significantly associated with the increased rates of HBeAg loss and HBeAg seroconversion.

**Table 3:** Impact of on-treatment HBV DNA on 5-year outcomes

	N	VR	P-value	HBeAg loss	P-value	HBeAg seroconversion	P-value
drop of HBV DNA ≥3 log_10_copies/ml at week 4	12	91.7%	0.06	91.7%	0.013	41.7%	0.083
drop of HBV DNA < 3 log_10_copies/ml at week 4	35	62.6%		51.4%		17.1%	
drop of HBV DNA ≥4 log_10_copies/ml at week 12	20	85.0%	0.366	80.0%	0.028	45.0%	0.006
drop of HBV DNA < 4 log_10_copies/ml at week 12	27	60.0%		48.0%		7.4%	
DNA level < 4 log_10_copies/ml at week 24	25	96.0%	< 0.001	84.0%	0.001	36.0%	0.030
DNA level ≥ 4 log_10_copies/ml at week 24	22	40.9%		36.3%		9.1%	
DNA negative at week 52	27	100%	< 0.001	88.9%	< 0.001	33.3%	0.062
DNA positive at week 52	20	45.0%		20.0%		10.0%	

### 5. Resistance to ADV

Four patients experienced viral breakthrough at week 104, 184, 208, 260 respectively. HBV DNA rebounded to 2.2 × 10^4^- 6.5 × 10^7 ^copies/ml, and ALT flares were found in 3 patients but none of them developped liver decompensation. ADV correlated mutations were detected in 3 of the 4 patients (1 of them with N236T and another 2 with combination of A181T/V and N236T mutations in HBV DNA polymerase). None of the patients who achieved HBV DNA < 4 log copies/ml at week 24 experienced viral breakthrough. All four patients were then given additional LAM for combination therapy.

### 6. Safety analysis

Four patients had an increase in serum creatinine of more than the peak value of normal (1.5 mg/dl), but the abnormality was not confirmed by a consecutive sample test. Three patients had slightly elevated serum uric acid in incontinuous samples. No action was taken regarding study medication dosing.

## Discussion

The response of ADV monotherapy for HBeAg positive CHB patients is less satisfying comparing with that of other oral NAs, but in many Asian countries like China, tenofovir is unavailable, ADV is still the only nucleotide analogue without cross resistance to other NAs to date [12-14]. The optimization of ADV therapy according to patients' baseline and on-treatment parameters may be a feasible strategy to increase the response. It was ever reported that ADV might provide additional benefits for HBeAg seroconversion in patients with pre-treatment HBV DNA levels between 10^7 ^and 10^8 ^copies/ml [15]. Subgroup analysis of GLOBE Study with telbivudine revealed that patients with ALT≥2 × ULN and HBV DNA < 10^9 ^copies/ml had more chance of VR, HBeAg seroconversion and ALT nomalization at year 2 [16]. There were also similar reports about long term LAM therapy [10]. In our study baseline factors such as ALT predicted long term response to ADV therapy. Patients with higher baseline ALT (≥5 × ULN) had more chance of optimal outcomes, and lower levels of HBeAg (< 800 s/co) and HBsAg(< 5000 IU/ml) also predicted HBeAg loss. In addition, there was a trend that patients with lower HBV DNA level might have higher rates of VR, HBeAg loss and HBeAg seroconversion although no statistical significance was observed in our study. Our findings may be limited by the relatively small sample size. When we combined baseline ALT and HBV DNA, we found that patients with baseline HBV DNA < 10^9 ^copies/ml and ALT ≥3 × ULN had more favorable outcomes. An elevated ALT level indicates increased clearance to HBV infected hepatocytes. It was reported that Chinese patients with ALT > 5 × ULN had high rate of spontaneous HBeAg seroconversion even without treatment [17], but treatment with anti-virus medicaments can improve the chance of HBeAg seroconversion and decrease the disease progression which has been proved in many clinical studies [2,18-20]. Similarly, HBeAg level may reflect the clearance capability of a HBV infected body to HBV infected hepatocytes [21,22]. The significance of HBsAg in treatment of patients with CHB is being paid close attention to recently. HBsAg may reflect the level of cccDNA in liver and the decrease during treatment with pegylated interferon alfa-2a may predict the sustained viral response [23,24]. More studies are needed to elucidate HBsAg variations during treament with NAs. In our study patients with lower level of baseline HBeAg and HBsAg had more chance of HBeAg loss which indicaties that baseline HBeAg and HBsAg may be regarded as subsidiary parameters in pretreatment evaluation.

Early HBV DNA reduction may also contribute to VR and HBeAg seroconversion in some studies [11,15,25]. It's generally accepted that therapy can be optimized by monitoring of serum HBV DNA levels during treatment with oral NAs and week 24 is the most important time point recommended to assess efficacy in LAM and telbivudine therapy [26], but whether it's reasonable in treatment of suboptimal antiviral drug like ADV remains controversial. We selected week 4, 12, 24 and 52 as time points in our study and found that serum HBV DNA < 4 log_10 _copies/ml at week 24 was predictive for VR, HBeAg loss and HBeAg seroconversion after 5-year treatment, The drop of HBV DNA from baseline ≥3 log_10 _copies/ml at week 4, ≥ 4 log_10 _copies/ml at week 12 and HBV DNA turning negative at week 52 can also predict favorable outcomes although not as optimal as HBV DNA < 4 log_10 _copies/ml at week 24. None of the patients who achieved HBV DNA < 4 log_10 _copies/ml at week 24 experienced viral breakthrough. Accordingly we recommend serum HBV DNA level < 4 log_10_copies/ml at week 24 as one of the major predictors for long term outcomes in patients with ADV therapy.

Our study has some limitations because of its small sample size and lack of a control group. However, in our study most patients completed 5 years' follow-up, and the detection method was advanced. The findings may give some evidence to manage HBeAg positive patients who intend to accept or have already accepted ADV therapy.

## Conclusions

ADV treatment may be initiated to patients with baseline ALT≥5 × ULN or patients with ALT≥3 × ULN and HBV DNA < 10^9 ^copies/ml, lower level of HBeAg(< 800 s/co) or HBsAg (< 5000 IU/ml) may be regarded as referenced factors. Patients will be followed up regularly and long term outcome may be predicted according to on-treatment HBV DNA. For patients with HBV DNA level < 10^4 ^copies/ml at week 24 therapy should continue and favorable outcomes may be achieved in 5 years or longer. For others with HBV DNA ≥10^4 ^copies/ml at week 24, some modifications for the therapeutic regimen such as addition or switch to another agent should be considered to enhance the long term response.

## List of abbreviations

ADV: Adefovir Dipivoxil; ALT: alanine aminotransferase; CHB: chronic hepatitis B; HBeAg: hepatitis B e antigen; HBV: hepatitis B virus; LAM: lamivudine; NAs: nucleos(t)ide analogues; ULN: upper limit of normal; VR: viral response.

## Competing interests

The authors declare that they have no competing interests.

## Authors' contributions

HL conceived of the study, participated the design, carried out the study and drafted the manuscript. DYG carried out laboratory work, FS and JYZ participated in following up of patients and data collecting. BL participated in the design of the study and performed the statistical analysis. LXM participated in the design and coordination and helped to draft the manuscript. All authors read and approved the final manuscript.
